# Interlayer Bonding Capability of Additively Manufactured Polymer Structures under High Strain Rate Tensile and Shear Loading

**DOI:** 10.3390/polym13081301

**Published:** 2021-04-15

**Authors:** Patrick Striemann, Lars Gerdes, Daniel Huelsbusch, Michael Niedermeier, Frank Walther

**Affiliations:** 1 Laboratory of Material Testing, University of Applied Sciences Ravensburg-Weingarten, Doggenriedstraße 42, D-88250 Weingarten, Germany; niedermeier@rwu.de; 2 Department of Materials Test Engineering (WPT), TU Dortmund University, Baroper Str. 303, D-44227 Dortmund, Germany; lars.gerdes@tu-dortmund.de (L.G.); daniel.huelsbusch@tu-dortmund.de (D.H.); frank.walther@tu-dortmund.de (F.W.)

**Keywords:** additive manufacturing, high-speed testing, interlayer tensile strength, material extrusion

## Abstract

Additive manufacturing of polymers via material extrusion and its future applications are gaining interest. Supporting the evolution from prototype to serial applications, additional testing conditions are needed. The additively manufactured and anisotropic polymers often show a weak point in the interlayer contact area in the manufacturing direction. Different process parameters, such as layer height, play a key role for generating the interlayer contact area. Since the manufacturing productivity depends on the layer height as well, a special focus is placed on this process parameter. A small layer height has the objective of achieving better material performance, whereas a larger layer height is characterized by better economy. Therefore, the capability- and economy-oriented variation was investigated for strain rates between 2.5 and 250 s^−1^ under tensile and shear load conditions. The test series with dynamic loadings were designed monitoring future applications. The interlayer tensile tests were performed with a special specimen geometry, which enables a correction of the force measurement. By using a small specimen geometry with a force measurement directly on the specimen, the influence of travelling stress waves, which occur due to the impact at high strain rates, is reduced. The interlayer tensile tests indicate a strain rate dependency of additively manufactured polymers. The capability-oriented variation achieves a higher ultimate tensile and shear strength compared to the economy-oriented variation. The external and internal quality assessment indicates an increasing primary surface profile and void volume content for increasing the layer height.

## 1. Introduction

**Figure 1:**
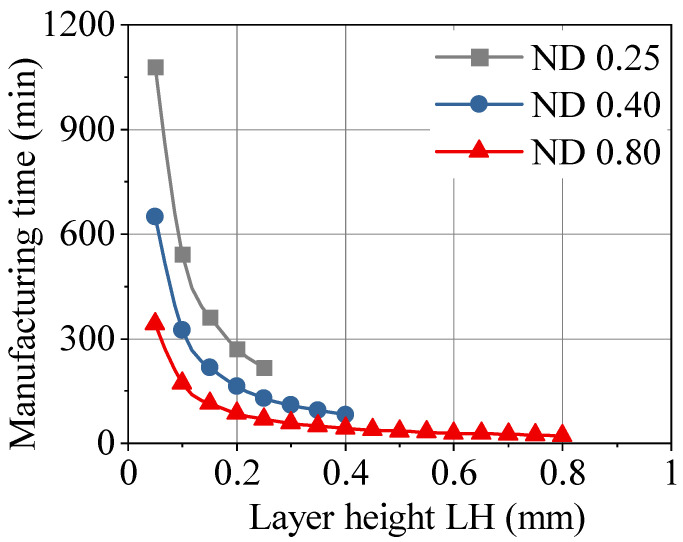
Manufacturing time vs. layer height depending on the nozzle diameter.

The application fields for extrusion-based additive manufacturing (AM) technologies are changing. The beginnings were dominated by prototyping or tooling, while the manufacturing of serial components has become a focus. In general, polymers and composites are used for their benefits, including being lightweight, corrosion resistant and economical [1]. Nevertheless, layer-based AM presents several challenges that must be addressed for a sophisticated manufacturing technology. This includes poorer mechanical properties and surface quality, and slow manufacturing speed compared to reference systems like injection molding [2,3,4]. Processing the same feedstock material results in reduced mechanical properties [5]. The material properties of the filament itself are less important, and the process–structure–property relationship results in the AM material properties. The layer-based structure yields anisotropic material behavior [6,7], while different effects of anisotropy are recognizable under quasi-static [8] and cyclic [9] loading. Compared to single-batched injection molded polymers, the fracture behavior is also highly anisotropic [10,11]. This strong dependency of AM polymers is due to the porosity and weld-lines [5], as well as the alignment of polymer molecules in an extrusion direction [12]. Therefore, the weakest point of an AM polymer—the interlayer area in manufacturing direction z—needs to be focused on in order to improve the overall mechanical properties [13,14]. For realistic application in series production, the surface quality is a major limiting factor, especially for functionality [15,16,17]. Therefore, the surface quality shows the same anisotropic behavior as the mechanical properties [18] and has a predictable shape, which is influenced by process parameters [2]. The process parameters nozzle diameter (ND) and layer height (LH) play a significant role in the surface quality, manufacturing time and part costs [19]. Combining these two process parameters with the extrusion velocity results in the manufacturing productivity [20]. Based on both experimental and simulated data, the relationship between ND, LH and manufacturing time is shown in Figure 1. In general, the ND serves as the limiting factor for the largest LH to be generated. An increase of LH leads to a substantial reduction in manufacturing time [21] and to rougher surfaces [22]. Therefore, the process–structure–property relationship of ND and LH merges conflicting goals and significant potential for process optimization. Unfortunately, the interactions of these process parameters are not fully understood, although the changes lead to multiple modifications of microstructure, mechanical properties and failure mechanism [23].

The interlayer material behavior of AM polymers is extremely brittle compared to the material behavior in other manufacturing directions or injection molding [24]. The mechanical properties for the interlayer contact area are improved by heating [25] or post-processing such as annealing [26]. In general, a smaller LH results in higher ultimate tensile strength and Young’s modulus [27]. In addition, the surface quality is optimized by smaller LH in terms of the staircase-effect and dimensional accuracy [28,29]. A larger LH leads to lower mechanical properties [27] and coarse surface qualities like increased waviness [28] or the staircase effect [2], but reduces the manufacturing time [19]. In this study, a classification is made from a capability- and economy-oriented variation. Within this work, a small LH of 0.2 mm, representing improved material performance, and a large LH of 0.3 mm, representing better productivity, are used.

These two application-oriented process variations were combined with application-driven testing conditions. Thus, the as-built surface texture, without post-processing, was tested. For monitoring future applications like crash-relevant components, the strain rate dependency of the interlayer contact area was investigated [30]. Under quasi-static loading, polymers are often subjected to a ductile failure, whereas a brittle failure occurs under dynamic loading [31]. The brittle failure for the interlayer contact area under quasi-static loading leads to the research demand of the material behavior under high strain rates. Therefore, investigation of the capability- and economy-oriented variation under different strain rates is necessary.

## 2. Materials and Methods

**Figure 2:**
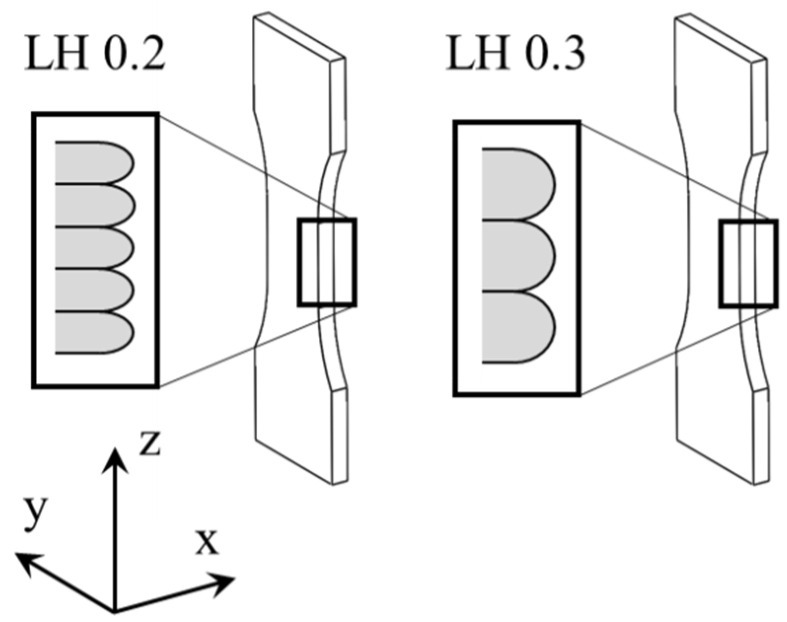
Manufacturing orientation and scheme for as built surface texture.

The experimental investigations were executed with single batch feedstock material. The short carbon fiber-reinforced polyamide (SCFRP) (CarbonX™ Nylon Gen.3, 3DXTECH, Grand Rapids, MI, USA) has a fiber weight content of approx. 12.5 wt.%, a carbon fiber diameter of 7 µm and a fiber length distribution between 150 and 400 µm. The extrusion-based additive manufacturing was done by a commercial system (Ultimaker 2+ Extended, Ultimaker, Geldermalsen, The Netherlands). The manufacturing orientation of specimens is visualized in Figure 2. Selected manufacturing parameters are given in Table 1. Variation is achieved by changing the LH, while the overall process parameters are kept constant. These modifications of LH lead to different macroscopic surface textures. Because of the application-oriented testing environment, no post processed surface treatment was performed. The manufacturing was done in standard atmosphere according to DIN 527-1 at 23 ± 2 °C and 50 ± 10% relative humidity.

### 2.1. Experimental Setup

**Figure 3:**
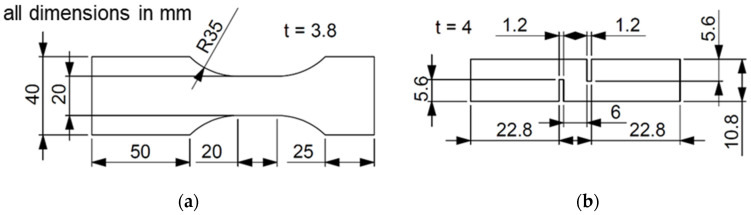
Specimen geometry for: (**a**) tensile tests and; (**b**) shear tests.

**Figure 4:**
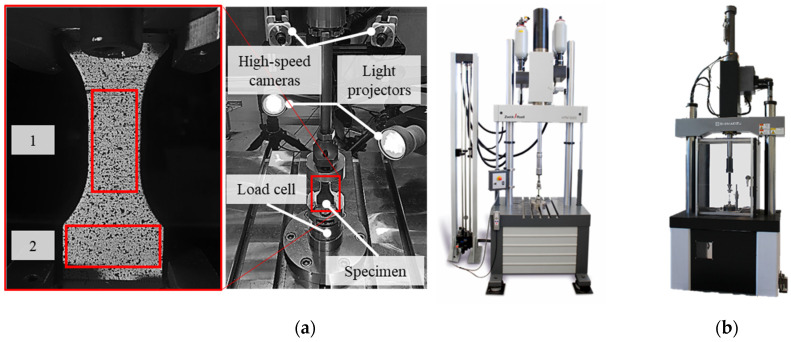
Experimental setup for high-speed tests: (**a**) HTM 5020 for tensile loading; (**b**) HITS-TX 10 for shear loading.

The experiments with dynamic tensile loading were done with the specimen geometry displayed in Figure 3a. This specimen geometry, based on DIN EN ISO 26203-2, was adapted with a one-sided extension of the clamping area in order to enable two sections for strain measurement. A combination of these two sections allows a correction of the measured force data due to retrospective recalculation. As given in the experimental setup in Figure 4a, the strain behavior was observed in the gage section (1) and the dynamometer section (2). The high-speed tensile tests were carried out on the servo-hydraulic high-speed testing system (HTM 5020, ZwickRoell, Ulm, Germany) achieving strain rates of 2.5, 25 and 250 s^−1^. The strain measurement was realized with the high-speed camera system (3D HHS, GOM, Braunschweig, Germany) with a recording rate of 64,000 fps at an image resolution of 256 × 256 pixels and a subsequent digital image correlation (DIC). The calibration of the high-speed camera system resulted in an error of 0.023 pixels, by an angle between the cameras of 25.2°. The high-speed shear tests were done with a high-speed testing system (HITS-TX 10, Shimadzu, Kyoto, Japan) while reaching a strain rate of 250 s^−1^. The displacement was monitored with a grip displacement sensor, which excludes non-specimen deformations from the measurement. The specimen geometry for shear tests is visualized in Figure 3b.

Detecting internal defects, the non-destructive quality assessment was carried out by a universal micro-computed tomography (µCT) inspection system (XT H 160, Nikon, Tokyo, Japan). The voxel size was set to 9 µm and thus detecting voids up to a minimum of 18 µm was possible. The external quality assessment in terms of surface texture was performed by 3D laser scanning confocal microscopy (VK-X100, Keyence, Osaka, Japan). The optical measurements were executed with a 10× lens and tilt correction by software application. No additional filters were used to further modify the visualization of surface texture. The resulting primary surface profiles were calculated using an averaged profile based on 100 measurements.

### 2.2. Data Processing

With the initial evaluation of high-speed tensile tests, stress-strain curves were obtained based on the measurements of global raw force data via load cell and local strain using DIC. When attaining high strain rates, stress waves within the load path often occur, which result in the system ringing effect, and thus superimposing the raw force data [32]. Therefore, the global force data have to be corrected to ensure comparable results for different strain rates. As shown in Figure 4, two local strain measurements at the gage section (1) and the dynamometer section (2) are realized. The dynamometer section (2) is specially designed for limiting the maximum strain to 0.25%. With the following assumptions, the global force data is corrected:

- No oscillations present at the beginning of the test since the stress wave travels a certain amount of time from the impact location until reaching the load cell.
- Identical stress-strain behavior in gage section (1) and dynamometer section (2).

**Figure 5:**
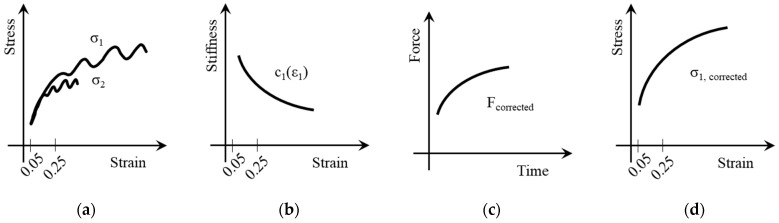
Schematic data processing for high-speed tensile testing: (**a**) stress vs. strain diagram based on raw data for gage and dynamometer section; (**b**) stiffness vs. strain within the gage section; (**c**) corrected force vs. entire test time; (**d**) corrected stress vs. strain in the gage section.

Figure 5 visualizes the data processing in schematic diagrams. At the beginning, no oscillations affect the testing system and the force data. As the test time increases, the force signal is continuously distorted by oscillations. Therefore, the calculation of stiffness within the gage section (1) is performed within the limits ε_t_ = 0.05 and 0.40% at the beginning of the tensile test with an unaltered force signal. In Figure 5b, the stiffness c, calculated via Hooke´s law, is presented.

c_1_(ε_1_) = σ_1_/ε_1_ (1)

The special design of the dynamometer section allows the recalculation of the force based on the stiffness c_1_ and local strain ε_2_ within the dynamometer section.

F_corrected_ = c_1_(ε_1_) × ε_2_ × A_2_ (2)

Assuming the influence of specimen geometry is negligible, test forces do not change in load direction. This applies to gage and dynamometer section as well as mounting and adapter components equally. Therefore, a retrospective recalculation of the stress distribution on the specimen, leading to corrected force F_corrected_, is possible. The corresponding cross section of gage section A_1_ is used to obtain the corrected stress in the gage section according to Equation (3).

σ_1, corrected_ = F_corrected_/A_1_ (3)

## 3. Results and Discussion

**Figure 6:**
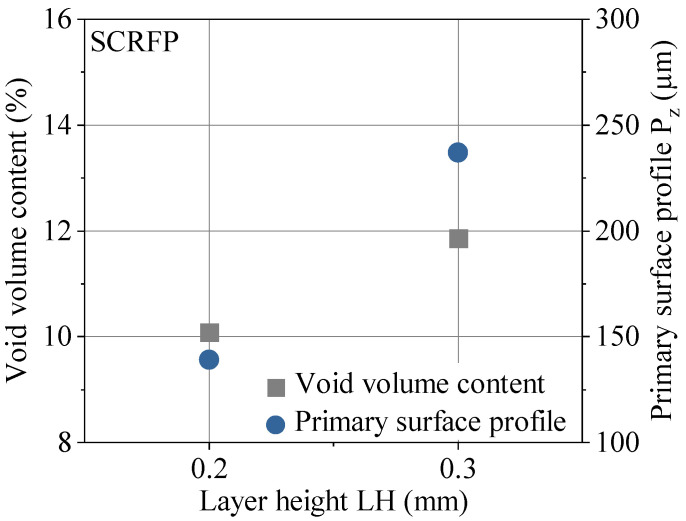
Results for the external and internal quality assessment via 3D laser scanning microscope and µCT.

**Figure 7:**
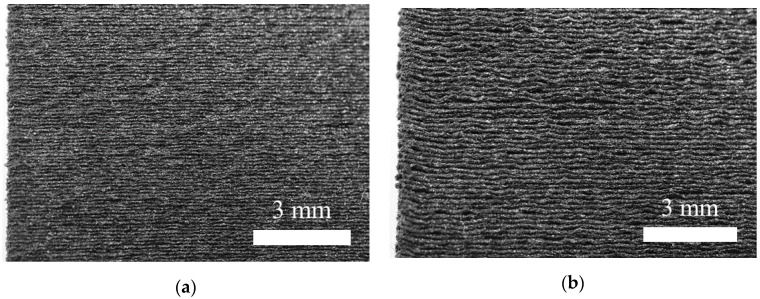
Macroscopic visualization of the manufacturing quality. (**a**) LH 0.2; (**b**) LH 0.3.

The results for the quality assessment are summarized in Figure 6. The non-destructive testing by µCT gives the internal defects of AM polymers. Manufacturing with higher LH leads to higher internal void volume content, which is consistent with previous studies [33]. The external quality assessment was executed using a 3D laser scanning microscope within the gage section (1). The results indicate an increasing primary surface profile by increasing LH. This finding confirms the current literature, in which the layer height has a direct influence on the surface quality in terms of waviness and roughness [2,28]. In order to specify the manufacturing quality, Figure 7 illustrates the original as built surface of the specimens for different LHs. The combination of the quantitative results of the primary surface profile and the visualization clarifies the difference in manufacturing quality for LH 0.2 mm and LH 0.3 mm.

**Figure 8:**
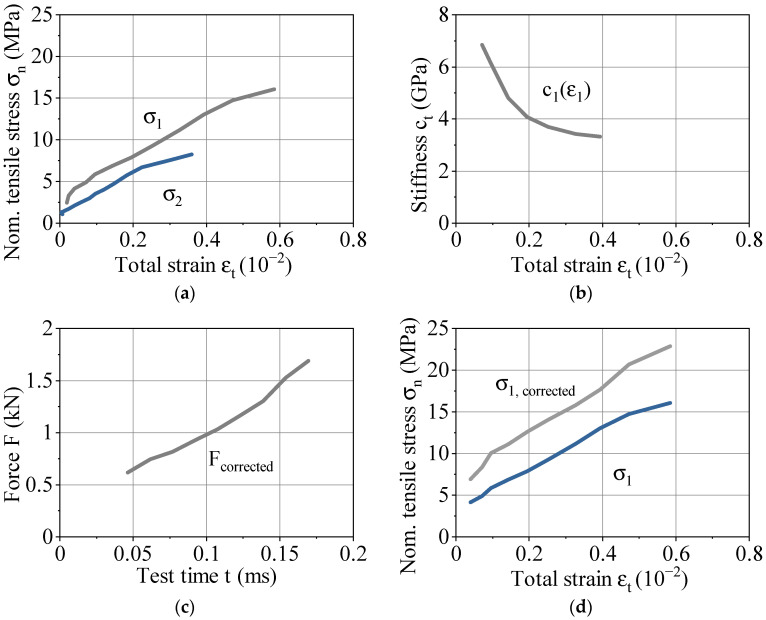
Exemplary data processing for high-speed tensile tests on representative data for LH 0.2 mm at a strain rate of 250 s^−1^: (**a**) stress vs. strain diagram with raw data including oscillations for testing and dynamometer section; (**b**) stiffness vs. strain out of the testing area; (**c**) corrected force vs. time for the entire test; (**d**) corrected stress vs. strain diagram in the testing area.

The raw data of high-speed tensile tests are processed according to the procedure in Figure 5. This data processing is exemplified on a representative test for LH 0.2 mm at a strain rate of 250 s^−1^ in Figure 8. The raw data of the experimental setup consists of synchronized stress-strain curves. Starting with a force signal, two stress-strain curves result for the gage section (1) and dynamometer section (2). The difference in slope in Figure 8a is in contradiction to Hooke´s law. The very beginning of the test is assumed to be unaffected by oscillations and stress waves and is therefore the basis for correction. The stiffness in the gage section (1) is calculated between 0.05% and 0.40% strain, resulting in Figure 8b. Combining this strain-resolved stiffness with the time-resolved stress in the dynamometer section (2) results in a corrected force of the entire test in Figure 8c. Using the corresponding cross sections, a corrected stress signal is calculated, as shown in Figure 8d.

The correction by two local strain measurements as described above combines benefits and challenges. The time-resolved, corrected force is based on idealized material behavior and an unaltered force signal at the beginning of the test, since there is no precise determination of the time when the stress waves start to influence the force signal. The maximum strain in the dynamometer section (2) in Figure 8 is about 0.40%. The correction of the total time-resolved force signal has to be done with the total strain-resolved signal in the dynamometer section. Thus, in this example, the assessment of stiffness must take place between 0.05% and 0.40% strain. Therefore, the correction procedure only serves as an approximation and the force signal is still affected. Optimization of the ratio between gage section (1) and dynamometer section (2) depending on the mechanical properties improves this error source. Enlarging the dynamometer section (2) for a lower maximum strain within this area can be described as an advantage for future experiments, since the determination of the curve in Figure 5b can be assigned to a reduced test time, thus excluding further oscillations.

**Figure 9:**
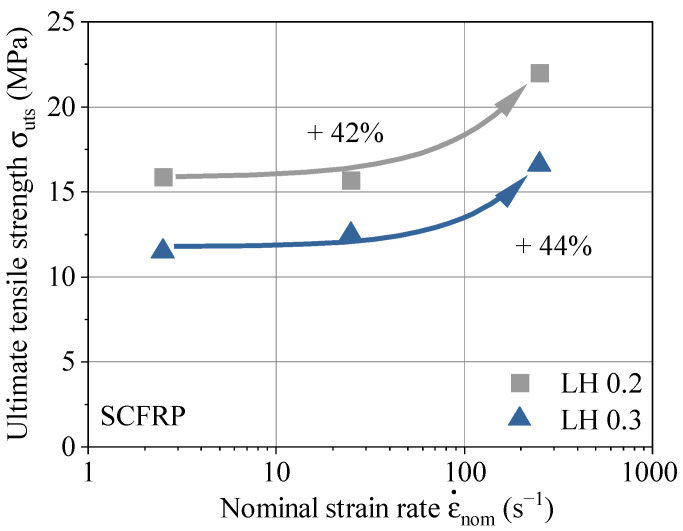
Ultimate tensile strength vs. strain rate for LH 0.2 and LH 0.3.

The results for all variations after the data processing are summarized in Figure 9. The diagram gives the ultimate tensile strength subjected to the nominal strain rate. Both LHs exhibit a positive strain rate dependency by an increase of ultimate tensile strength of 42% for LH 0.2 and 44% for LH 0.3 within the range of the examined strain rates. This material behavior is consistent with previous literature [34]. However, the increase in ultimate tensile strength is often accompanied by a decrease in strain [31]. The experimental study with dynamic tensile loading was designed for testing the interlayer tensile strength of the AM polymer. This type of loading behaves brittle under quasi-static conditions [24]. Therefore, the decrease in strain is less significant compared to the increase in ultimate tensile strength.

Within the test series, the smaller LH 0.2 achieves the higher ultimate tensile strength over the entire range of strain rates. The degradation of material capability with increasing LH under quasi-static loading conditions is well known [35], the material behavior with increasing strain rate is introduced with this work. The strength reinforcement tendency due to higher strain rates is considered as almost identical between 2.5 and 250 s^−1^ for LH 0.2 and LH 0.3.

**Figure 10:**
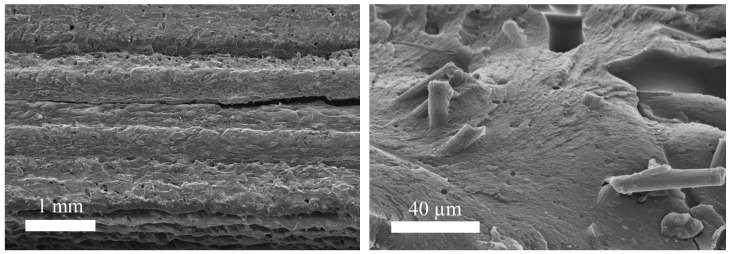
The fracture surfaces for LH 0.3 at a strain rate of 250 s^−1^.

The investigations of fracture behavior indicate the main failure mechanisms within the test series under tensile loading. Figure 10 shows an exemplary fracture surface of the specimen with LH 0.3 mm tested at a strain rate of 250 s^−1^. The fracture surface with low magnification indicates matrix cracking and interlayer debonding. The matrix cracking occurs both between and within extrusion beads. Failure between extrusion beads in manufacturing direction z is equivalent to an interlayer debonding. The fracture surface with high magnification demonstrates the additional failure mechanism of fiber pull-out. These failure mechanisms under high strain rates are consistent with current literature [36]. Furthermore, the internal quality is represented by visible voids.

**Figure 11:**
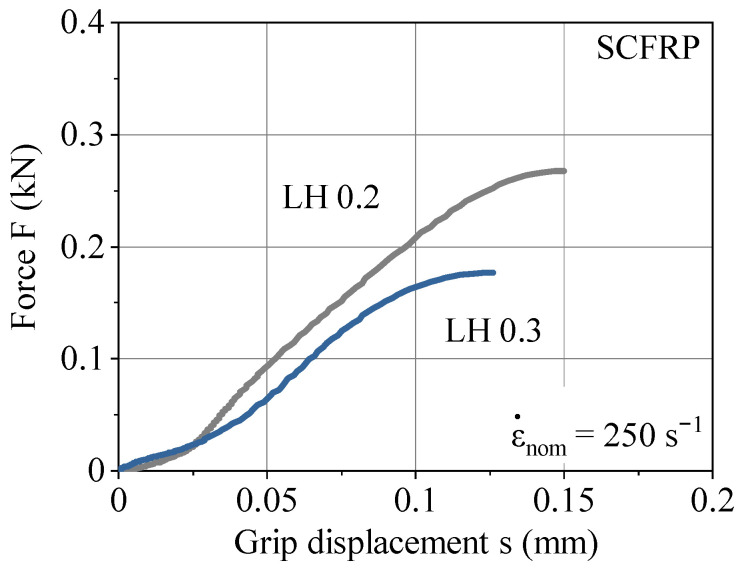
Force vs. grip displacement for LH 0.2 and LH 0.3 at a strain rate of 250 s^−1^.

The high-speed tests with shear loadings were performed at a strain rate of 250 s^−1^. The exemplary strain rate was chosen for a first comparison of different LHs under dynamic shear loadings. The specimen geometry in Figure 3b, using the high-speed testing system Shimadzu HITS-TX, enables a displacement measurement via a grip sensor. Therefore, no retrospective data processing for this experimental setup was necessary. The force vs. displacement curves in Figure 11 show no obvious oscillations. Furthermore, the tests provide the shear strength τ_uts_ of 11.1 MPa for LH 0.2 and 7.4 MPa for LH 0.3. This indicates a reduction of shear strength under dynamic loading of about 30% due to the increasing LH, which is equal to the reduction of shear strength under quasi-static loading [33]. Thus, it can be said that the strain rate is not affecting LHs differently, but instead affects the overall strength. This applies to the tests under tensile and shear loading equally.

## 4. Conclusions

For the successful advancement of the material extrusion to a serial production process, application-oriented testing conditions are required. Additive manufacturing by material extrusion results in layer-based and highly anisotropic material with an obvious weakness in the interlayer contact area. This interlayer material behavior is defined by process–structure–property relationships. Due to the significant influence of the process parameter layer height (LH) on the material performance and the manufacturing productivity, the interlayer bonding capability was focused on in this study. Based on the LH, two conflicting goals for serial production are affected. The small LH results in better material capability, the larger LH leads to decreasing manufacturing time. Therefore, the capability- and economy-oriented variation is considered under application-oriented conditions.

For this, a short carbon fiber-reinforced polyamide (SCFRP) was investigated with strain rates between 2.5 and 250 s^−1^. The experimental setups were designed for focusing on the interlayer contact area with tensile and shear loadings. The special design of the dynamic tests under tensile loading ensures a retrospective recalculation of force data. Micro-computed tomography and 3D laser scanning confocal microscopy were used for quality assessment for the capability- and economy-oriented variation.

The quality assessment highlights that the void volume content and the primary surface profile increase with higher LH. The results of dynamic tests under tensile loading show a strain rate dependency of SCFRP. A smaller LH leads to higher ultimate tensile strength for all strain rates. The trend of strength reinforcement due to higher strain rates is almost the same for LH 0.2 and 0.3. The results with dynamic shear loading highlight an increased shear strength with decreasing LH. The test series expands the process knowledge for material extrusion, in particular the process–structure–property relationship based on the LH. Regarding future applications, these findings are valuable for the design of serial components manufactured by material extrusion.

In further investigations, the test series will be intensified with additional strain rates and LHs. In consideration of nozzle diameter, an additional process parameter will be included in the process–structure–property relationship. This enables a more precise characterization of the capability- and the economy-oriented variation. Furthermore, with extended testing conditions, monitoring for applications will be in a wider range. The experimental setup for dynamic tensile tests will be adapted through additional design iterations. Modifying the specimen geometry will reduce the maximum strain in the dynamometer section (2) and the corresponding error for the retrospective calculated force data.

## Figures and Tables

**Table 1:** Selected manufacturing parameters for material extrusion process in Simplify 3D.

Parameter	Unit	CarbonX™
Nozzle diameter (ND)	mm	0.4
Extrusion bead	mm	0.5
Layer height (LH)	mm	0.2 and 0.3
Extrusion temperature	°C	260
Printing bed temperature	°C	80
Extrusion velocity	mm·s^−1^	10
Specimen orientation	-	ZYX
Infill percentage	%	100
Raster pattern	-	unidirectional 0°
Number of contours	-	1

## Data Availability

The data presented in this study are available on request from the corresponding author.
